# Inconsistent Intersample ALK FISH Results in Patients with Lung Cancer: Analysis of Potential Causes

**DOI:** 10.3390/cancers12071903

**Published:** 2020-07-14

**Authors:** Zhenya Tang, Hui Chen, Lingzhi Hong, Guilin Tang, Gokce A. Toruner, Wei Wang, Sinchita Roy Chowdhuri, Wei Yin, Hai Suk Jung, Jun Gu, Mark J. Routbort, Jianjun Zhang, Joseph D. Khoury, L. Jeffrey Medeiros

**Affiliations:** Departments of Hematopathology, Pathology and Thoracic/Head and Neck Medical Oncology, School of Health Professions, The University of Texas MD Anderson Cancer Center, 1515 Holcombe Blvd., Houston, TX 77030, USA; HChen7@mdanderson.org (H.C.); LHong@mdanderson.org (L.H.); GTang@mdanderson.org (G.T.); GAToruner@mdanderson.org (G.A.T.); WWang13@mdanderson.org (W.W.); SRoy2@mdanderson.org (S.R.C.); WYin1@mdanderson.org (W.Y.); HJung2@mdanderson.org (H.S.J.); jungu@mdanderson.org (J.G.); mark.routbort@mdanderson.org (M.J.R.); JZhang20@mdanderson.org (J.Z.); JKhoury@mdanderson.org (J.D.K.); ljmedeiros@mdanderson.org (L.J.M.)

**Keywords:** lung cancer, ALK, fluorescence in situ hybridization (FISH), next-generation sequencing (NGS), RNA sequencing (RNA-seq), tyrosine kinase inhibitor (TKI)

## Abstract

ALK FISH analyses of multiple specimens occasionally yield inconsistent intersample results in lung cancer patients, posing clinical challenges requiring intensive analysis of all potential causative pre- and post- analytic factors. In this study, 19 patients (8M/11F) with inconsistent intersample ALK FISH results were analyzed, representing 4.9% of patients assessed ≥ twice in our institution. Fifteen patients received ALK tyrosine kinase inhibitor(s) (TKIs). Nine patients died, and ten were alive for 8 to 74-month follow-ups (median, 40 months). Through strict and stringent laboratory and case-review policies, all postanalytic factors were excluded. Correlating clinical information, ALK results obtained by RNA sequencing (RNA-seq) and other concurrent tests, several pre-analytic factors were determined. A suboptimal specimen was likely the cause in three patients, supported by the failure of one or more concurrent tests or discrepant results between FISH and RNA-seq. ALK inhibition by TKIs might have been responsible for the change of *ALK* status from positive to negative in eight patients. Other potential explanations include the existence of multiple primary lung cancer lesions, tumor heterogeneity, and the clonal evolution of tumor cells, related or not to ALK TKI therapy. This study is helpful for both pathologists and clinicians encountering inconsistent and/or discrepant intersample results.

## 1. Introduction

Approximately 3–7% of nonsmall cell lung cancer (NSCLC) patients have neoplasms with constitutive anaplastic large-cell lymphoma kinase (ALK) activity due to ALK abnormalities, most frequently in the form of intrachromosomal inversion and consequent ALK rearrangement with the partner echinoderm microtubule associated protein-like 4 (EML4) forming EML4-ALK fusion. Crizotinib, a first-in-class ALK tyrosine kinase inhibitor (TKI) [1], and all the other ALK TKIs such as certinib [2], alectinib [3], lorlatinib [4] and brigatinib [5] have shown significant effects in improving both progression-free survival and overall survival in patients with ALK positive lung cancer in the past decade. Several new candidate ALK inhibitors, as well as combinations of existing ALK inhibitors, have also shown great promise in clinical trials [6,7,8]. The most important indication for ALK TKI administration, as well as the definitive predictive marker for a potential good response, is the ALK rearrangement in the tumor tissues. Therefore, unambiguous identification of ALK status in a lung cancer specimen plays a vital role in the clinical management of these patients. Multiple assays that have been developed and applied for the assessment of ALK status, including fluorescence in situ hybridization (FISH), immunohistochemistry (IHC), polymerase chain reaction (PCR) and reverse-transcription PCR (RT-PCR) assays, and next generation sequencing (NGS)-based DNA sequencing (DNA-seq) and RNA sequencing (RNA-seq) technologies. In numerous studies, improvements and optimization of these assays, rational diagnostic algorithms utilizing some or all these assays, and potential causes and solutions for the discrepant results obtained by different assays [9,10,11,12] have been reported.

More than one lung cancer specimen from the same patient over time may be collected for ALK testing. The two major reasons for sequential testing are the evaluation of newly detected neoplasms (either recurrent or relapsed primary tumors, or metastatic tumors) and monitoring treatment response. Inconsistent ALK results (positive to negative or vice versa) in different tumor specimens from the same patient can occur; this phenomenon was designated as “intersample-discrepant results” by Lambros et al. [13]. Theoretically, inconsistent ALK status can be caused by many pre- and post- analytical factors. The occurrence of inconsistent results can be a challenge for pathologists interpreting the test results and clinicians making decisions for patient management, such as starting an ALK TKI, adjusting the dose or discontinuing an ALK TKI, or switching to a different therapeutic strategy.

In this retrospective study, we report 19 lung cancer patients who had inconsistent intersample ALK FISH results obtained from multiple tumor samples collected at different time points during their clinical courses. The aim of this study was to better understand the frequency and causes of inconsistent ALK FISH results in lung cancer patients.

## 2. Results

### 2.1. Patients and General Information

**Figure 1:**
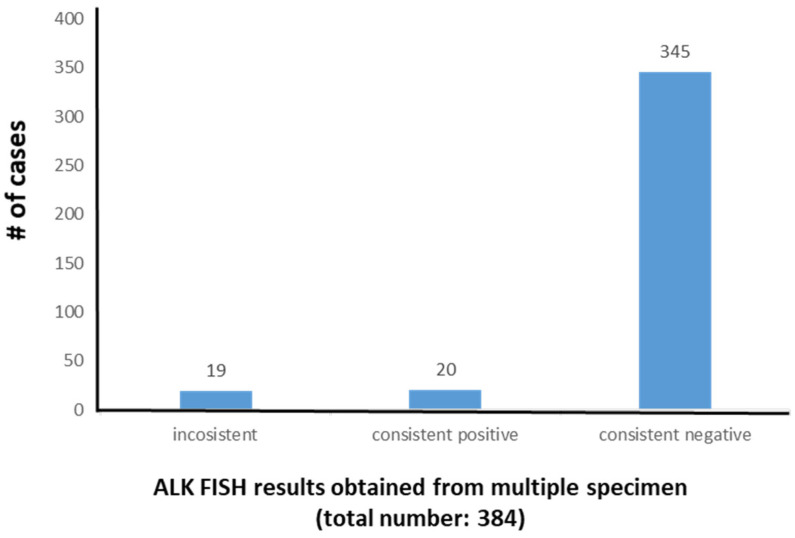
ALK FISH results of multiple specimens collected at different time points in 384 lung cancer patients.

In the tissue FISH database of the Clinical Cytogenetics Laboratory during the time interval of this study, 384 lung cancer patients were tested two or more times for ALK FISH. Nineteen (4.9%) of these patients had tumor samples that exhibited inconsistent intersample ALK FISH results (positive to negative or vice versa). The remaining patients had consistent, i.e., either positive (*n* = 20, 5.2%) or negative (*n* = 345, 89.9%), ALK FISH results (Figure 1). The 19 patients with inconsistent results formed the study group.

There were 8 men and 11 women with a median age of 59 years (range, 29 to 73 year). These patients received various types of therapy based on their clinical condition, such as surgery, radiation, gamma-knife and targeted therapy with EGFR inhibitor(s), VEGF inhibitor(s) and/or PD-1 inhibitor(s). Fifteen (79%) patients also received at least one type of ALK TKI. One patient (case #5) did not receive ALK TKI due to renal insufficiency, a known contraindication. Three patients (cases #3; #13 and #14) were treated with other targeted therapies, such as gefitinib, Osimertinib, durvalumab and bevacizumab, and two of them (cases #13 and #14) already achieved good responses without ALK TKI, whereas one (case #3) died shortly after ALK FISH testing (Table 1). Three patients had a history of other types of tumors before their lung cancer (case #4 had thyroid cancer; cases #5 and #7 had non-Hodgkin lymphomas). Of note, case #15 had a history of large cell neuroendocrine carcinoma and adenocarcinoma involving the right upper lobe of the lung, diagnosed in another hospital. With a median follow-up/survival of 40 months (range, 8 to 74 months), nine patients died and ten were alive at the last follow-up.

### 2.2. ALK FISH and Other Laboratory Findings 

**Figure 2:**
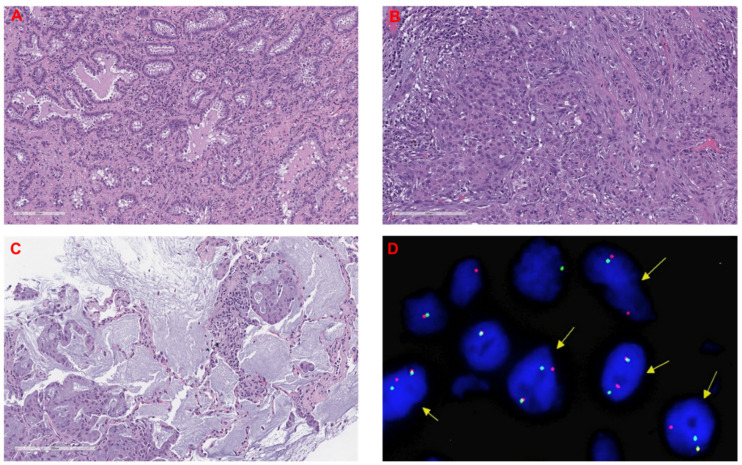
Representative pathological features of lung cancer. (**A**) Moderately differentiated adenosquamous carcinoma (from the first specimen of case #18); (**B**) Squamous cell carcinoma (from the second specimen of case #18); (**C**) Adenocarcinoma with mucinous differentiation and signet ring cell features (from the third specimen of case #19); and (**D**) ALK FISH image. Arrows indicate ALK rearrangement positive cells for typical split signal patterns (from the third specimen of case #19) (Magnification: 10X for histology; 100X for FISH).

A total of 44 tumor specimens collected from these 19 patients at different time points was assessed (Table 2). Sixteen (84.2%) patients had lung adenocarcinoma with various features, such as good, moderate or poor differentiation, and mucinous and/or signet ring cells. Two (10.5%) patients (cases #7 and #9) had adenocarcinoma in one tumor specimen and squamous cell carcinoma in another tumor specimen. One patient (5.3%) (case #18) had adenosquamous carcinoma in his first tumor specimen and squamous cell carcinoma in his two subsequent tumor specimens (Figure 2). In nine patients (cases #1, #4, #6-#8, #10, #11, #16 and #18), samples were collected before and after ALK TKI administration, whereas in the remaining ten patients, all samples were collected before ALK TKI administration. Thirteen patients had two sample tested and six had three samples tested, In 15 patients, at least one sample tested was the primary lung cancer with the other sample being a recurrent lung tumor or a metastasis (lymph node, pericardial fluid, liver, brain). In case #1, all three tumor samples were collected from the left lung but at different time points (primary vs. relapsed vs. metastatic lung cancers). In case #6, both samples were collected from liver metastases, but one prior to and the other after ALK TKI administration. It should be mentioned that two samples (the first sample in cases #2 and #11) were collected from pericardial fluid but prepared differently in this study. The first sample of case #2 was a cell block section prepared from cytospin, and the first of case #11 was a cytology smear preparation.

ALK tissue FISH analyses were performed on 42 tumor samples, but not on the second samples of two patients (cases #18 and #19). A total of 21 (50%) samples were positive for *ALK* rearrangement, with positive result rates ranging from 18 to 88% (median, 50%) of nuclei. One or more concurrent FISH tests for *RET*, *ROS1* and/or *MET* were performed on 30 samples. Two patients (cases #4 and #10) were positive for *MET* amplification and one (case #6) was positive for *ROS1* rearrangement. Thirty patients had a concurrent NGS-based DNA-seq assay performed and 25 (83.3%) had at least one mutation detected (Table 2).

An RNA-seq assay was implemented for clinical services in our institution in 2018. Eight tumor samples from five patients in this cohort were tested by RNA-seq, and five (62.5%) were positive for *EML4-ALK* fusion; all of these samples were collected before ALK TKI administration. However, a discrepancy between ALK FISH and RNA-seq was observed in three out of six samples (cases #12, #13 and #18). The first specimen of case #12 was tested twice with ALK FISH, but only 9–10% of tumor cells were identified as positive for *ALK* rearrangement, which is below the established cutoff value (≥15%) for a positive result. RNA-seq in this patient was positive for *EML4-ALK* fusion. The discrepancy can likely be explained by sensitivity; the number of *ALK* rearrangement positive cells in this specimen was below the limit of detection by FISH, but not by RNA-seq. The second specimen collected approximately one year later from the same patient was positive by ALK FISH and RNA-seq. The second specimen of case #13 was low positive (22%) by ALK FISH but negative by RNA-seq. This specimen was obtained from a para-esophageal lymph node by fine needle aspiration (FNA) biopsy. NGS DNA-seq detected multiple gene mutations, including EGFR amplification, that were not detected by RNA-seq. Here, we need to point out that our RNA-seq platform (Oncomine) is an amplicon-based assay, detecting the most common ALK fusions, such as *EML4-ALK*, *KIF5B-ALK*, and *HIP1-ALK*. An *ALK* fusion with a rare or a novel partner gene may not be detected by this assay, whereas the ALK FISH assay detected ALK rearrangement, regardless of the partner gene. The first specimen of case #18 was positive by ALK FISH (86%), but RNA-seq failed due to inadequate RNA. This specimen was submitted from another hospital, and only limited material was available. The second sample collected from this patient approximately 3 months later was *EML4-ALK* fusion positive by RNA-seq (Table 2). Further investigation of other potential causes for the discrepant results in these three patients (cases #12, #13 and #18) are indeed necessary, but our efforts in this respect have been hampered by the unavailability of remaining material of these samples.

The causes for the discrepancy between ALK FISH and RNA-seq observed in this study were most likely the lower sensitivity of ALK FISH versus NGS-based RNA-seq (e.g., case #12), and a lack of an adequate specimen/positive cells available for RNA isolation (e.g., cases #13 and #18). Other researchers have provided similar explanations for discrepant results [14,15].

## 3. Discussion

The goals of this study were to assess the frequency and potential causes of inconsistent ALK FISH results in consecutive samples of patients with lung cancer. From a group of 384 patients with lung cancer tested two or more times by FISH to assess ALK status, we observed inconsistent intersample results in about 5% of patients. The remaining patients tested had intersample concordant results, with approximately 5% concordant positive and 90% concordant negative. The high frequency of negative results was expected, as we test all new patients with lung cancer by FISH for *ALK* status, and the frequency of *ALK* rearrangement in lung cancer ranges from 3 to 7% in various studies [1,16]. To the best of our knowledge, the results in this study are novel and helpful for clinical application. To date, only one research group has reported discrepant intersample results for ALK status in lung cancer patients [13]. Lambros et al. compared ALK expression detected by IHC to ALK FISH results in18 lung cancer patients with two or more tumor samples tested; the authors identified intersample discrepancies in eight patients. Since all the samples in their study were collected before ALK TKI administration, their study focused mainly on the comparison of ALK IHC and ALK FISH methods for diagnostic purposes, as well as the potential biologic mechanism(s) for the discrepancies derived from two different methods [13].

All 19 patients in the current study exhibited inconsistent results in a series of lung cancer samples, many of which were collected from different anatomic sites and at different time points. This phenomenon can pose clinical management challenges, both for the pathologist interpreting the results and for the physician managing the patient. Inconsistent test results may cause confusion regarding the diagnosis, and could influence the selection of treatment, evaluation of response, and assessment of prognosis. Therefore, an extensive investigation of all pre- and post- analytical factors that can potentially explain inconsistent results may be helpful.

Through a strict policy of consensus between two or more pathologists for all difficult cases, including those with discrepant results, potential postanalytical causes for the inconsistent ALK FISH results in this study have been excluded. By following stringent laboratory policies for inclusion of several negative and positive controls during each run of testing, and re-analyzing any samples with discrepant results by two additional, independent approaches, analytical factors as an explanation for inconsistent results also can, in large part, be excluded in this study.

Several pre-analytical factors may explain the inconsistent intersample ALK FISH results in this cohort (Table 2). The quality and/or quantity of some specimens were suboptimal for testing, e.g., body fluids or small samples collected by fine needle aspiration (FNA) biopsy sometimes provide only limited material for testing, supported by the fact that some of the ordered tests failed in these samples (Table 2). The age of each tumor tissue sample (interval between sample collection and testing) was also analyzed. Approximately 60% of specimens were tested for ALK FISH and other concurrent tests within 1 week, and about 75% of samples were tested within 2 weeks of sample collection. The remaining 10 samples were tested from 21 to 1156 days after being obtained, but all yielded reliable test results (Table 2). Therefore, it seems that the age of the specimen did not impact the ALK FISH results in this study [14,15].

The intervals between sample collection and their relationship to ALK TKIs (before vs. after) are included in Table 2. Sample collection prior to or after ALK TKI administration may have had an impact on the inconsistent intersample ALK status in some of these cases. In eight patients, the ALK status changed from positive in the first tested sample to negative in a subsequent sample. This change may indicate that ALK TKIs are effective in eliminating ALK rearranged tumor cells. These results also suggest that therapeutic agents other than ALK TKIs may be needed at the time ALK FISH becomes negative. The subsequent *ALK* rearrangement negative tumor cells may be related to clonal evolution under selective pressure by ALK TKIs. In one patient (case #1), the initial sample was negative, but the second and third samples were positive for ALK rearrangement by FISH. The cause for this discrepancy remains unknown. Of interest, the second sample was collected prior to ALK TKI administration, and the third was collected 1 month after ALK TKI administration; ALK FISH signals were reduced by approximately 50% after ALK inhibition. This individual is still disease-progression free (Table 1).

Ten cases in this study exhibited inconsistent intersample ALK FISH results in their tumor tissues collected before ALK TKI administration, but from different anatomic sites. Lambros et al. [13] had similar observations in five out of eight lung cancer patients in their study. In correlation with all clinical information, including the anatomic sites of sample collection, the inconsistent ALK FISH results may be attributable to the complexity of lung cancer that can present in multiple forms, such as multiple primary lung cancer lesions, or may reflect intra- and inter- tumor heterogeneity, as can be manifested as distinct tumor morphologies, genetic profiles, and response to various therapies [17,18,19,20]. For example, two different histological types of lung cancer were observed in different specimens in two patients (cases #7 and #9). In case #7, adenocarcinoma was present in the initial specimen collected from level 4R lymph node biopsy specimen, and squamous cell carcinoma was present in the second retroperitoneal biopsy specimen. In case #9, squamous cell carcinoma was diagnosed in the first cervical lymph node biopsy specimen, and adenocarcinoma was diagnosed in the subsequent retroperitoneal lymph node biopsy specimen. Therefore, these two patients were likely harboring tumors with mixed histologic types, and their ALK statuses were compared between two histologic types of cancer, perhaps explaining the discrepancy. Another patient (case #18) presented initially with moderately differentiated adenosquamous carcinoma in the first biopsy specimen collected in his right lung lesion, which was ALK positive by FISH. The second specimen, collected later from a recurrent lesion in his right main bronchus, showed a squamous cell carcinoma and was positive for ALK rearrangement, as assessed by RNA-seq. His third and most recent tumor biopsy specimen collected from his left lung pathologically showed a minute focus of squamous cell carcinoma that was negative for ALK rearrangement by FISH (Table 2, Figure 2). Overall, these discrepant results suggest that gene status varies among tumor tissues that are collected from different anatomic sites, or even the same anatomic site at different time points, further supporting speculation that there is a mixture of subclones in lung cancers. These subclones can have distinct morphological and genetic features, including a different ALK status, and may further evolve, independent of or in response to therapies such as ALK inhibition. This phenomenon has an important practical implication, i.e., that tumor specimens collected from different anatomic sites or even the same anatomic site but at different time points in a lung cancer patient may need to be tested for ALK status [9,12,13].

The updated ALK testing guidelines from the College of American Pathologists, the International Association for the Study of Lung Cancer, and the Association for Molecular Pathology emphasize that both ALK immunohistochemistry and NGS-based methods are alternatives to ALK FISH for the detection of ALK status and selection of lung cancer patients for ALK TKI administration [21]. Prior to the new guidelines published in 2018, ALK FISH was considered the gold standard for testing ALK status [22]. In this retrospective study, most specimens (36/44) were collected before 2018, and ALK status was assessed by ALK FISH only. The remaining specimens collected in 2018 and later, especially those with a negative ALK FISH result, were also evaluated by RNA-seq. As we observed, the test results of RNA-seq can be affected by multiple pre-analytic factors, such as the quality/quantity of the specimen and the presence of rare types of ALK rearrangement [10,11,23,24]. Therefore, an alternative method, e.g., IHC or RT-PCR, even if impractical for clinical laboratories, may be needed to further investigate ALK status in some patients [9,25,26].

## 4. Materials and Methods

### 4.1. Case Selection

We searched the tissue FISH database of the Clinical Cytogenetics Laboratory at The University of Texas MD Anderson Cancer Center from 1 November 2012 to 31 March 2020. This time frame corresponds to the interval during which the United States Food and Drug Administration (FDA, Silver Spring, MD, USA) approved the Vysis ALK Break Apart FISH Probe Kit (Abbott Molecular, Des Plaines, IL, USA) for assessments of lung cancer specimens. Lung cancer patients who were tested at least twice, but with inconsistent ALK FISH results, were included in this study. A chart review was performed in which we recorded clinical and laboratory information including dates of diagnosis and testing, methods used for obtaining specimens (e.g., surgical biopsy vs. fine needle biopsy, FNB), places of sample collection (in our hospital vs. outside hospital), age of specimen (the interval between dates of sample collection and FISH testing), treatments and response/outcomes. This study was approved by the Institutional Review Board (IRB protocol# PA14-0693), and was conducted by following institutional guidelines with informed consent in accordance with the Declaration of Helsinki.

### 4.2. ALK Tissue Fluorescence In Situ Hybridization (FISH)

As reported previously, formalin-fixed, paraffin-embedded (FFPE) tissue sections of lung cancer specimens and the FDA-approved Vysis ALK Break Apart FISH Probe Kit were used to assess ALK status [9,16]. Following the guidelines provided by the vendor, 50 nuclei were analyzed routinely for all specimens (50-cell analysis), but analyses were extended to 100 nuclei (100-cell analysis) if a result from the first 50-cell analysis was indeterminate. The following criteria for a positive ALK rearrangement were suggested by the vendor, and also validated in our laboratory: >25 cells of the first 50-cell analysis, or >15 cells of the 100-cell analysis that exhibit typical split signal patterns for ALK rearrangement [27,28]. Tissue FISH tests for other markers related to lung cancer, such as RET, ROS1 or MET, were also performed. The probe and procedures for these markers were reported previously [16]. According to our laboratory policy, at least two well-trained for tissue FISH and certified technologists participated in the analysis/counts of each specimen. If a discrepancy of ≥10% between two technologists occurred, a third technologist was involved in the analysis until the discrepancy was resolved. Any specimen with a failure of results, an indeterminate result, an inconsistent result with previous sample(s) of the same individual, or a discrepant result to that obtained by another method was re-analyzed or repeated by two or more technologists so that a consensus among the technologists participating in the testing was reached. For all slides tested, a FISH probe was applied to the region(s) in the FFPE slide checked and labelled by pathologists to avoid regions with low tumor cellularity and/or tumor necrosis. According to our established criteria for inclusion/exclusion of clinical specimens for testing, any specimen with <60% of tumor cellularity and/or apparent tumor necrosis was rejected for testing.

### 4.3. NGS-Based DNA Sequencing for Detection of Gene Mutations and Gene Amplifications in Tumor Tissues

The Pico Pure DNA Extraction Kit (Arcturus, Mountain View, CA, USA) and the Agentcourt AMPureXP Kit (Agentcourt Biosciences, Beverly, MA, USA) were employed for DNA purification from marked areas of FFPE tumor sections from each specimen (usually five unstained slides with a minimum of 20% tumor cellularity). Approximately 10 ng (50-gene panel) or 20 ng (134-gene panel) of purified DNA were applied for targeted NGS-based DNA-seq for detections of gene mutations and gene amplifications, as reported previously [10,11,29,30]. DNA sequencing was performed using the Ion Torrent PGM, but the number of target genes was historically expanded from 50 (Ion AmpliSeq Cancer Hotspot Panel, Thermo Fisher Scientific, Waltham, MA, USA) to 134 (OncoMine Comprehensive Assay, Thermo Fisher Scientific), referred to as the “50-gene panel” and “134-gene panel”, respectively [10,11,29,30]. However, genes such as ALK, BRAF, EGFR, KRAS, RET and many other mutations that are considered to be hotspots for lung cancer are included in both 50-gene and 134-gene panels. DNA extraction from peripheral blood of the same individual (in most patients) was used as a control for the differentiation of constitutional and somatic aberrations in tumor specimens.

### 4.4. NGS-Based RNA Sequencing (RNA-Seq) for Detection of Gene Fusions in Tumor Tissues

As part of the OncoMine Comprehensive Assay, NGS-based RNA-seq was included for targeted inter- and intra- genic fusions involving 51 clinically important genes, including the common EML4-ALK fusion for lung cancer [11]. The FormaPure Kit (Agentcourt Biosciences, Beverly, MA, USA) was employed for RNA purification from FFPE tumor tissues. Approximately 10 ng of RNA for each specimen was utilized for fusion detection. This RNA-seq assay is amplicon-based and specific for EML4-ALK fusions. It was validated and implemented for clinical services in our Molecular Diagnostic Laboratories [11] (please see File S1 and S2 for a list of genes for somatic mutation detection included in the gene panel, and a list of gene fusions detected by RNA-seq).

## 5. Conclusions

In this study, we show that inconsistent intersample ALK FISH results occur in about 5% of lung cancer patients. In large part, we can exclude analytical or postanalytical factors as the cause(s), as our processes avoid these errors as explained above. Instead, we suggest multiple potential explanations for the intersample inconsistency of ALK status. In about 15% of the patients in this cohort, pre-analytic factors may have been responsible as some of the specimens analyzed by FISH were suboptimal. In another third of the patients in this cohort, positive ALK FISH results led to treatment with ALK TKI and a subsequent specimen was negative by FISH for ALK rearrangement. Therapeutic efficacy may explain this subgroup of inconsistent results. Lastly, in about half of these patients, the explanation for inconsistent intersample results is not clear but may be attributable to differences in the biologic features inherent in relapsed or metastatic lung cancer specimens compared with the primary tumor, for example, tumor heterogeneity or clonal evolution. Therapy can also influence clonal evolution. We believe that the results in this study are practically helpful for both pathologists and physicians encountering inconsistent intersample ALK FISH results as well as discrepant results obtained by ALK FISH and RNA-seq.

## Figures and Tables

**Table 1:** General information on lung cancer patients in this study.

General Information						Interventions									
Case #	M/F	Age (y) *	D/A	FU ** (m)	Specimen #	Chemo	Others	ALK TKIs and Responses ***							
								Crizotinib	Response	Alectinib	Response	Ceritinib	Response	Brigatinib	Response
1 ^	M	58	A	74	1	Yes	surgery								
					2										
					3			Yes	CR	Yes	CR				
2	F	29	A	41	1	No	gamma-knife								
					2			Yes	PD (toxicity)						
3	M	57	D	40	1	Yes	radiation								
					2										
4 ^^	F	69	A	27	1	Yes	radiation, sugery								
					2										
					3					Yes	PD in brain				
5 ^^^	F	73	D	17	1	No	radiation, sugery								
					2										
6	F	65	D	41	1	No	gamma-knife	Yes	PR in lung and liver, PD in bone						
					2										
7 ^^^	M	41	D	12	1	Yes	No	Yes	D						
					2										
8	F	60	A	50	1	No	No	Yes	PD	Yes	PD	Yes	SD, toxicity	Yes	PD in brain
					2										
9	M	54	D	20	1	Yes	erlotinib								
					2			Yes	PD, toxicity						
10	F	68	D	32	1	No	radiation	Yes	PR to PD						
					2										
					3										
11	M	61	D	8	1	No	No	Yes	PR to PD	Yes	PD				
					2										
					3										
12	F	31	A	25	1	Yes	radiation								
					2					Yes	CR				
13	M	61	A	45	1	Yes	surgery, gamma knife; durvalumab								
					2										
14	F	32	A	45	1	Yes	bevacizumab								
					2										
15 ^+^	F	67	D	67	1	Yes	radiation, surgery								
					2			Yes	PD						
16	M	62	D	17	1	Yes	surgery	Yes	PD in LN			Yes	PD	Yes	PD
					2										
17	F	59	A	73	1	Yes	a heghog inhibitor and pembrolizumab	Yes	PD in LN			Yes	PD	Yes	PD
					2										
18	M	47	A	23	1	Yes	sugery, bevacizumab							Yes	PR in right lung; PD in left lung
					2										
					3										
19	F	56	A	51	1	Yes	sugery, radiation, Entrectinib								
					2										
					3									Yes	not yet

M/F: male/female; D/A: dead/alive; Chemo: chemotherapy; y: year; m: month; PD: progressive disease; SD: stable disease; PR: partial response: CR: complete response; * Age at initial diagnosis of lung cancer; ** Follow up/survival: between dates of initial diagnosis of lung cancer to the last visit or death; *** ALK TKI agent administered after collection/testing of that specimen; ^ Primary tumor in right lung; ^^ History of thyroid cancer; primary tumor lesion at left lung; ^^^ History of non-Hodgkin lymphoma; + History of RUL large cell neuroendocrine carcinoma and adenocarcinoma; Specimen#: following the dates of sample collection; ALK TKI administration after collection of the specific sample.

**Table 2:** Information on tumor tissues, test results and potential causative causes for inconsistent intersample ALK FISH results.

General Information of Tumor Tissues							Results of Various Tests				Potential Causes for Inconsistent Results		
Case #	Test# by Dates	Origin of Tumor Tissues *	Pathologic Diagnosis	Age of Samples (d) **	Intervals (d) ***	ALK TKIs^	ALK FISH Result (%)	Other Tissue FISH Tests	Gene Mutations	RNA-Seq ^^	Associated with ALK TKIs	Primary vs. Metastasis	Tumor Cmplexity
1	1	lung, LLL	adenocarcinoma, moderately differentiated	21	0	before	neg	*ROS1-*	ND	ND	Likely	No	Likely
	2	lung, LLL, lobectomy	adenocarcinoma, well-differentiated	77	44	before	56	*ROS1-, MET-*	neg	ND			
	3	lung, LUL	adenocarcinoma, with mucinous feature	9	552	after	30	*ROS1-, RET-*	*SMAD4:c.1082G>A*	ND			
2	1	fluid, pericardial	adenocarcinoma, metastatic	0	0	before	neg	*ROS1-*	ND	ND	No	Yes	Likely
	2	lung, right, FNA	adenocarcinoma	9	125	before	29	ND	ND	ND			
3	1	lung, LUL	adenocarcinoma	7	0	before	33	*RET-*	*EGFR:c.2573T>G; TP53:c.706T>C*	ND	Yes	Yes	UN
	2	liver	adenocarcinoma, metastatic	14	626	before	neg	*ROS1-*	*EGFR:c.2573T>G,c.2369C>T; TP53:c.706T>C*	ND			
4	1	lung, RML	adenocarcinoma, poorly differentiated	0	0	before	neg	*MET+, ROS1 and RET inconclusive*	*TP53:c.818G>A*	ND	Yes	No	Likely
	2	brain, right frontal	adenocarcinoma, metastatic, poorly differentiated	0	761	before	22	*MET+, ROS1-*	ND	ND			
	3	brain, right occipital	adenocarcinoma, metastatic, poorly differentiated	0	1034	after	neg	ND	ND	ND			
5	1	lung, left	adenocarcinoma with mucinous features	0	0	before	54	*MET-, ROS1-,RET-*	*FGFR2:c.755C>T*	ND	No	UN	Likely
	2	lung, right	adenocarcinoma	274	35	before	neg	*MET-, ROS1-,RET-*	ND	ND			
6	1	liver	adenocarcinoma, metastatic	32	0	before	50	*MET-, ROS1+ (outside ALK-, ROS1+)*	*EGFR-, KRAS-, BRAF- (outside)*	ND	Yes	No	Likely
	2	liver	adenocarcinoma, metastatic	7	172	after	neg	ND	ND	ND			
7	1	LN, Level 4R	adenocarcinoma with signet ring cell features, metastatic	0	0	before	72	*MET-, ROS1-, RET-*	*CSF1R:c.895G>A*	ND	Yes	No	Likely
	2	mass, retroperitoneal	squamous cell carcinoma, metastatic	0	254	after	neg	ND	ND	ND			
8	1	lung, RLL	adenocarcinoma	0	0	before	88	*MET-, ROS1-, RET-*	neg	ND	Yes	Yes	Likely
	2	fluid, pericardial	adenocarcinoma, metastatic	0	78	after	neg	ND	ND	ND			
9	1	LN, left cervical	adenocarcinoma, metastatic, p63 neg	73	0	before	neg	*MET-, ROS1-, RET-*	*TP53:c.736_742del*	ND	No	No	Very likely
	2	LN, retroperitoneal	squamous cell carcinoma, metastatic, p63 pos	0	405	before	18	*MET-, ROS1-, RET-*	*TP53:c.736_742del*	ND			
10	1	LN, FNA	adenocarcinoma	0	0	before	30	*ROS1-, RET-*	*EGFR:c.2573T>G*	ND	Yes	No	Likely
	2	Lung, RUL	adenocarcinoma	0	377	after	26	*MET-, ROS1-, RET-*	*EGFR:c.2573T>G,c.2369C>T; MDM2 amp*	ND			
	3	Lung, RUL	adenocarcinoma	0	676	after	neg	*MET+, ROS1-, RET-*	*EGFR:c.2573T>G, MET, CDK4 and MDM2 amp*	neg			
11	1	fluid, pericardial	adenocarcinoma, metastatic	0	0	before	19	*MET-, ROS1-, RET-*	*KRAS:c.35G>T; TP53:c.743G>T; MYC amp*	ND	Yes	Likely	Likely
	2	lung, LUL	adenocarcinoma	0	218	after	neg	ND	ND	ND			
	3	bone, T10	adenocarcinoma, metastatic	0	231	after	neg	ND	*KRAS:c.35G>T; TP53:c.743G>T; MYC amp*	ND			
12	1	mass, neck	adenocarcinoma, metastatic, poorly differentiated	21	0	before	neg	*MET-, ROS1-, RET-*	*TP53:c.916C>T*	*pos*	No	Likely	Likely
	2	LN, left mediastinum	adenocarcinoma, metastatic	0	371	before	54	ND	*TP53:c.916C>T*	pos			
13	1	lung, RUL, lobectomy	Adenocarcinoma, solid and acinar	0	0	before	neg	*ROS1-, RET-*	*TP53:c.481G>T*	ND	No	Yes	Yes
	2	LN, paraesophageal, FNA	adenocarcinoma, metastatic, poorly differentiated	0	911	before	22	*MET-, ROS1-, RET-*	*TP53:c.481G>T, ATRX:c.4517G>A, FANCD2:c.3055C>A, PDGFRB:c.1006C>A; POLE:c.2284C>T, RAD50:c.205G>T, EGFR amp*	neg			
14	1	Lung, LUL	Adenocarcinoma, PD-L1 neg	14	0	before	neg	*MET-, ROS1-, RET-*	neg	ND	No	Yes	Yes
	2	liver	adenocarcinoma, metastatic, poorly differentiated, PD-L1 pos	0	1270	before	56	ND	*BRCA1:c.4255G>T; FANCA:c.2890C>G*	pos			
15	1	LN, 4R, FNA	adenocarcinoma, metastatic	0	0	before	neg	*MET-, ROS1-, RET-*	*TP53:c.380C>G p.S127C*	ND	No	Likely	Likely
	2	lung, LLL	adenocarcinoma	0	5	before	68	ND	ND	ND			
16	1	LN, 4L, FNA	adenocarcinoma	0	0	before	26	*ROS1-, RET-*	*NRAS:c.181C>A; TP53:c.856G>T*	ND	Yes	No	Likely
	2	LN, left retroperitoneal	adenocarcinoma, metastatic	0	286	after	neg	*ROS1-*	*CREBBP:c.2626G>A; ERBB4:c.3699del; FGF19:c.33G>A; FLT3:c.942T>G; NRAS: c.181C>A; RAD50:c.370G>T; TP53: c.856G>T*	ND			
17	1	mass, mediastinal	adenocarcinoma, poorly differentiated	295	0	before	21	*MET-, ROS1-, RET-*	*NRAS:c.182A>T; TP53:c.479_480delinsAT; SMO:c.1174G>T*	ND	No	Yes	Likely
	2	LN, left supraclavicular	adenocarcinoma, metastatic	14	454	before	neg	*RET-*	*NRAS:c.182A>T; TP53:c.479_480delinsAT; SMO:c.1174G>T*	ND			
18	1	lung, RUL, lobectomy	adenosquamous carcinoma, moderately differentiated	480	0	before	86	ND	neg	inadequate RNA	Yes	Likely	Likely
	2	lung, right main stem bronchus	squamous cell carcinoma	28	28	before	ND	ND	neg	pos			
	3	lung, LUL	minute foci of squamous cell carcinoma	14	150	after	neg	ND	ND	ND			
19	1	LN, station 7, FNA	adenocarcinoma, metastatic	14	0	before	neg	*MET-, ROS1-, RET-*	neg	ND	No	Yes	Likely
	2	lung, LLL	adenocarcinoma with mucinous differentiation and signet ring cell features	1156	2275	before	ND	ND	*SETD2:c.6322_6333delinsC; SF3B1:c.1859T>C*	pos			
	3	lung, RLL	adenocarcinoma with mucinous features	7	1466	before	58	*MET-, ROS1-, RET-*	ND	ND			

d: days; FNB: fine needle biopsy; LN: lymph node; neg: negative; pos: positive; ND: not performed; amp: amplification; LLL: left lower lobe; LUL, left upper lobe; RLL: right lower lobe; RML: right middle lobe; RUL: right upper lobe * sampling through surgical biopsy, unless it’s noted as “FNB”. The 1st sample of case #2 was cytoblock section prepared from pericardial fluid, and the 1st sample of case #11 was cytology smear prepared from pericardial fluid; ** interval between sample collection and FISH testing; *** interval from the collection date of first specimen; ^ Before or after administration of ALK TKIs; ^^ EML4-ALK fusion.

## Supplementary Materials

The following are available online at https://www.mdpi.com/2072-6694/12/7/1903/s1, File S1: Gene list for somatic mutation and/or copy number variations (CNVs), File S2: List of gene fusions.
