# Supplementary material for: Inconsistent Intersample ALK FISH Results in Patients with Lung Cancer: Analysis of Potential Causes
Source: Cancers (Basel). 2020 Jul 14;12(7):1903. doi: 10.3390/cancers12071903 (PMC7409019; doi:10.3390/cancers12071903)
Supplement: Supplementary file 1 [file cancers-12-01903-s001.pdf]

Supplementary Materials:

Inconsistent Intersample ALK FISH Results in Patients with Lung Cancer: Analysis of Potential Causes

Zhenya Tang Hui Chen, Lingzhi Hong, Guilin Tang, Gokce A. Toruner, Wei Wang, Sinchita Roy Chowdhuri, Wei Yin, Hai Suk Jung, Jun Gu, Mark J. Routbort, Jianjun Zhang, Joseph D. Khoury and L. Jeffrey Medeiros

File S1. Gene list for somatic mutation and/or copy number variations (CNVs). The somatic mutations in the coding sequence of 134 genes and selected copy number variations (amplifications) in 47 genes (overlap: 146 genes in total listed below) are included in the platform.

AKT1	*BTK*	*CREBBP*	*FGF19*	*HRAS*	*MAPK1*	*NBN*	*PIK3CB*	*RAF1*	*SPOP*
AKT2	*CBL*	*CSF1R*	*FGF3*	*IDH1*	*MAX*	*NF1*	*PIK3R1*	*RB1*	*SRC*
AKT3	*CCND1*	*CTNNB1*	*FGFR1*	*IDH2*	*MDM2*	*NF2*	*PMS2*	*RET*	*STAT3*
ALK	*CCND2*	*DDR2*	*FGFR2*	*IGF1R*	*MDM4*	*NFE2L2*	*POLE*	*RHEB*	*STK11*
AR	*CCND3*	*EGFR*	*FGFR3*	*JAK1*	*MED12*	*NOTCH1*	*PPARG*	*RHOA*	*TERT*
ARAF	*CCNE1*	*ERBB2*	*FGFR4*	*JAK2*	*MET*	*NOTCH2*	*PPP2R1A*	*RICTOR*	*TOP1*
ARID1A	*CDK12*	*ERBB3*	*FLT3*	*JAK3*	*MLH1*	*NOTCH3*	*PTCH1*	*RNF43*	*TP53*
ATM	*CDK2*	*ERBB4*	*FOXL2*	*KDR*	*MRE11A*	*NRAS*	*PTEN*	*ROS1*	*TSC1*
ATR	*CDK4*	*ERCC2*	*GATA2*	*KIT*	*MSH2*	*NTRK1*	*PTPN11*	*SETD2*	*TSC2*
ATRX	*CDK6*	*ESR1*	*GNA11*	*KNSTRN*	*MSH6*	*NTRK2*	*RAC1*	*SF3B1*	*U2AF1*
AXL	*CDKN1B*	*EZH2*	*GNAQ*	*KRAS*	*MTOR*	*NTRK3*	*RAD50*	*SLX4*	*XPO1*
BAP1	*CDKN2A*	*FANCA*	*GNAS*	*MAGOH*	*MYC*	*PALB2*	*RAD51*	*SMAD4*	
BRAF	*CDKN2B*	*FANCD2*	*H3F3A*	*MAP2K1*	*MYCL*	*PDGFRA*	*RAD51B*	*SMARCA4*	
BRCA1	*CHEK1*	*FANCI*	*HIST1H3B*	*MAP2K2*	*MYCN*	*PDGFRB*	*RAD51C*	*SMARCB1*	
BRCA2	*CHEK2*	*FBXW7*	*HNF1A*	*MAP2K4*	*MYD88*	*PIK3CA*	*RAD51D*	*SMO*	

File S2. List of gene fusions. A set of targeted fusion sequences corresponding to clinically relevant known inter- and intragenic fusions in the following 51 genes can be detected by the platform used in this study.

AKT2, ALK, AR, AXL, BRAF, BRCA1, BRCA2, CDKN2A, EGFR, ERB84, ERBB2, ERG, ESR1, ETV1, ETV4, ETV5, FGFR1, FGFR2, FGFR3, FGR, FLT3, JAK2, KRAS, MDM4, MET, MYB, MYBL1, NF1, NOTCH1, NOTCH4, NRG1, NTRK1, NTRK2, NTRK3, NUTM1, PDGFRA, PDGFRB, PIK3CA, PPARG, PRKACA, PRKACB, PTEN, RAD51B, RAF1, RB1, RELA, RET, ROS1, RSPO2, RSPO3, and TERT

© 2020 by the authors. Licensee MDPI, Basel, Switzerland. This article is an open access article distributed under the terms and conditions of the Creative Commons Attribution (CC BY) license (http://creativecommons.org/licenses/by/4.0/).
